# Ultra-broadband Kerr microcomb through soliton spectral translation

**DOI:** 10.1038/s41467-021-27469-0

**Published:** 2021-12-14

**Authors:** Gregory Moille, Edgar F. Perez, Jordan R. Stone, Ashutosh Rao, Xiyuan Lu, Tahmid Sami Rahman, Yanne K. Chembo, Kartik Srinivasan

**Affiliations:** 1 Joint Quantum Institute, NIST/University of Maryland, College Park, MD USA; 2 Microsystems and Nanotechnology Division, National Institute of Standards and Technology, Gaithersburg, MD USA; 3 Institute for Research in Electronics and Applied Physics, University of Maryland, College Park, MD USA

**Keywords:** Nanophotonics and plasmonics, Nonlinear optics, Microresonators

## Abstract

Broadband and low-noise microresonator frequency combs (microcombs) are critical for deployable optical frequency measurements. Here we expand the bandwidth of a microcomb far beyond its anomalous dispersion region on both sides of its spectrum through spectral translation mediated by mixing of a dissipative Kerr soliton and a secondary pump. We introduce the concept of synthetic dispersion to qualitatively capture the system’s key physical behavior, in which the second pump enables spectral translation through four-wave mixing Bragg scattering. Experimentally, we pump a silicon nitride microring at 1063 nm and 1557 nm to enable soliton spectral translation, resulting in a total bandwidth of 1.6 octaves (137–407 THz). We examine the comb’s low-noise characteristics, through heterodyne beat note measurements across its spectrum, measurements of the comb tooth spacing in its primary and spectrally translated portions, and their relative noise. These ultra-broadband microcombs provide new opportunities for optical frequency synthesis, optical atomic clocks, and reaching previously unattainable wavelengths.

## Abstract (web summary)

Integrated optical frequency measurements, benefit from broadband on-chip frequency combs. Here the authors present a low-noise microcomb whose span extends from telecom to near-visible wavelengths. Here the authors present a dissipative Kerr soliton formation approximated by introducing the concept of synthetic dispersion.

## Introduction

Microresonator frequency combs are promising for chip-scale metrology applications including coherent range measurements^1^, spectroscopy^2^, and optical clocks^3,4^. These applications are typically realized in the dissipative Kerr soliton (DKS) regime of microcomb operation^5^, and often rely on stabilization of the comb repetition rate and carrier-envelope offset frequency, the latter usually through a *f*-2*f* interferometer^6,7^. *f*-2*f* stabilization requires at least an octave of comb bandwidth, which can be achieved through geometric dispersion engineering^8,9^ to create coherent dispersive waves (DWs) that broaden the comb spectrum^3,10–13^. Although DWs significantly increase microcomb bandwidth, the power of these enhanced comb teeth is still orders of magnitude lower than the pump, so that the *f*-2*f* technique remains challenging. For example, an end-to-end comb bandwidth of one octave is insufficient for self-referencing using the (centrally located) high-power pump. More complicated resonator cross-sections^14^ and stacks of different materials^15,16^ have been proposed to alter the dispersion in support of ultra-broadband combs, yet remain to be demonstrated experimentally. Other approaches for super-octave microcomb generation include combining *χ*^(2)^ and *χ*^(3)^ effects^17^, but such broadband combs usually present spectral gaps^18^, and the suitability of such combs^17,19,20^ for metrology has not been shown.

Here, we present a low-noise microcomb whose span extends across 1.6 octaves—without spectral gaps—while bridging the telecom with near-visible wavelengths. This is made possible through dual pumping, in which the second pump enables the *χ*^(3)^ process of four-wave mixing Bragg scattering (FWM-BS)^21–23^ to significantly broaden the typical DKS state, by spectral translation of the soliton into other spectral bands. Using the dual-pump scheme, we demonstrate that the DKS teeth, acting as the signal in the FWM-BS process, can be translated to new frequencies and effectively create new DWs on both sides of the original DKS spectrum, broadening its bandwidth by more than a factor two. The parametric nature of the FWM-BS process is such that phase coherence is expected to be maintained, which we probe experimentally through a series of noise measurements. In particular, heterodyne beat notes across the spectrum, measurements of the comb tooth spacing in both the original DKS portion and the spectrally translated portion, and a measurement of the relative noise between the comb teeth in the overlap between the two microcomb portions, are all consistent with the picture that FWM-BS spectrally translates the soliton—thereby preserving its repetition rate—to the spectral region surrounding the second pump, and the resulting 1.6 octave comb operates in a low-noise state. The incorporation of the FWM-BS spectral translation mechanism allows for a tunability and engineering of new DWs well beyond the limits imposed by geometric and material dispersion on conventional singly pumped microresonator DKS states. To better understand the potential of this system, we introduce the new concept of synthetic dispersion, which captures the underlying physics and predicts the comb behavior as a function of resonator geometry and pump frequencies. Simultaneously, we perform a detailed numerical study using a single multi-pump Lugiato–Lefever Equation^24,25^ that accounts for the full set of *χ*^(3)^ processes occurring in the resonator and validates the novel concept of synthetic dispersion. The synthetic dispersion framework is further validated by close correspondence with our experimental measurements of ultra-broadband microcombs created by FWM-BS spectral translation of a DKS state.

## Results

### Spectral translation and the synthetic dispersion framework

**Fig. 1:**
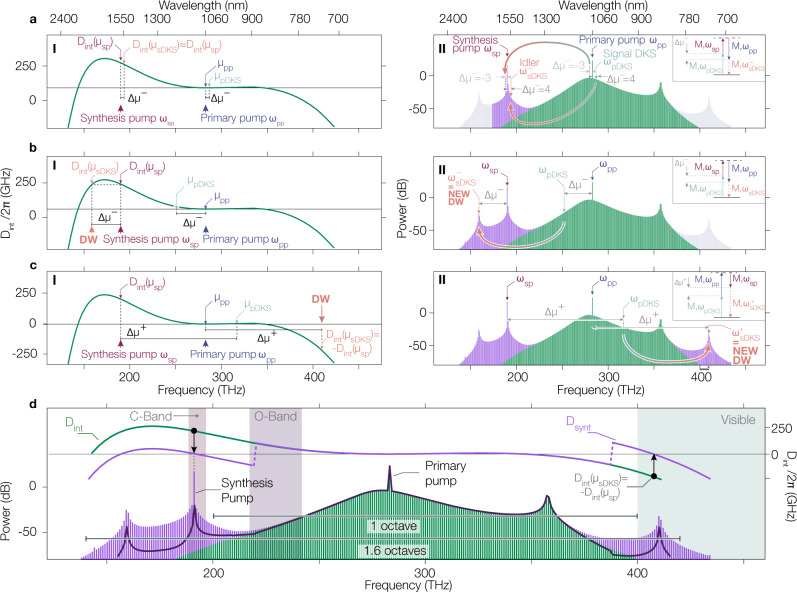
Soliton spectral translation and synthetic dispersion. **a** Integrated dispersion *D*_int_ (panel I) of a ring resonator with two zero-crossings. Under a single-pump (primary pump *pp*) drive, only one DW at high frequency is created due to power considerations (panel II). This part of the spectrum is referred to as the primary portion. Inclusion of another pump, the synthesis pump (sp), allows for four-wave mixing Bragg scattering (FWM-BS), especially considering the negative (lower frequency) idler process (panel I) translating comb teeth surrounding *μ*_pp_ to spectral positions surrounding *μ*_sp_, creating a synthesized portion of the frequency comb (panel II, purple spectrum). **b** The same negative idler frequency and momentum conservation condition is met for larger mode separation Δ*μ*, as the higher order dispersion coefficients allow for a roll-off of the integrated dispersion, and therefore another mode at the same integrated dispersion value as at the synthesis pump exists (panel I) and creates a new dispersive wave (DW) at low frequency (panel II). **c** Now considering the higher frequency idler due to FWM-BS (panel I), the frequency matching condition now imposes *D*_int_(*μ*_sp_) = − *D*_int_(*μ*_*sds*_), and therefore a DW in the primary portion of the comb must already exist (e.g., at 355 THz) to allow a change of sign of *D*_int_ (panel I). Here, this frequency matching condition is met twice, but the mode matching condition permits only the high-frequency mode to undergo FWM-BS. In the same manner as previously, it effectively creates a new DW at high frequency (at 410 THz), extending the frequency comb bandwidth on this side of the spectrum (panel II). **d** Introduction of a synthetic dispersion *D*_synt_ (purple, right axis) that captures the nature of the translated portions as new DWs at low and high frequencies (solid green, right axis). The resulting LLE simulated comb spectrum with a single pump (green) and a dual pump (purple) exhibit a clear difference of bandwidth. The synthetic integrated dispersion emulates the dual-pump system by an effective single-pump system (dark purple line) and shows close agreement with the dual-pump simulation. 0 dB is referenced to 1 mW, i.e., dBm.

A microresonator’s integrated dispersion *D*_int_ represents the variation of the cold cavity resonance frequencies away from an equidistant frequency grid (i.e. the DKS comb teeth) spaced by *D*_1_/2*π*, the free spectral range (FSR) around the primary pump^26^, hereafter annotated with the label *pp*. ${D}_{{{{{{{{\rm{int}}}}}}}}}(\mu )={\omega }_{{{{{{{{\rm{res}}}}}}}}}(\mu )-\omega$_DKS_$(\mu )={\omega }_{{{{{{{{\rm{res}}}}}}}}}(\mu )-({\omega }_{{{{{{{{\rm{pp}}}}}}}}}+{D}_{1}\mu)$, with *μ* defined as the mode order relative to the pumped mode *μ*_pp_ = 0 (i.e. *D*_int_(*μ*_pp_) = 0), ${\omega }_{{{{{{{{\rm{res}}}}}}}}}(\mu )$ being the cavity resonance frequency of the mode *μ*, and *ω*_DKS_(*μ*) being the *μ*^th^ DKS comb tooth frequency. Hence, when the cavity resonances match the DKS comb teeth frequencies, i.e., *D*_int_(*μ*_DW_) = 0, a resonant enhancement happens, leading to the DW creation at the mode position *μ*_DW_ (Fig. 1a). In this case (and for the microresonators we study below), *D*_int_ is such that only one primary DW ($DW^{\prime}$) is created, as the second zero crossing (on the low frequency side) is too far from the primary pump to yield appreciable energy.

The use of an auxiliary pump enables straightforward access to soliton states, through an effective temperature compensation mechanism that bypasses the thermal bistability^27–29^. Moreover, simultaneous spectral broadening of the comb, attributed to cross-phase modulation (XPM) effects, has also recently been observed^30^, though the magnitude of the effect was limited. Here, we consider a dual-pumped system in a regime where much more significant spectral broadening is realized. We pinpoint the origin of the strong increase in comb bandwidth as originating from an interband FWM-BS process. FWM-BS is mediated by the combination of a strong, secondary pump (hereafter referred to as the synthesis pump *sp*) and the primary pump *pp*, and results in phase-coherent spectral translation of comb teeth across wide spectral gaps determined by the difference in pump frequencies. While FWM-BS of a single frequency continuous wave input has been demonstrated in a microcavity^23^, and its role in soliton-DW mixing in the context of optical fibers has previously been studied in intraband^22^ and interband cases^21^, here we show how it can play a critical role in the creation of ultra-broadband microresonator frequency combs.

We consider a FWM-BS framework where the signal can be any comb tooth of the primary soliton (*pDKS*) resulting from the primary pump *pp* (i.e., the comb that would be obtained through a single pump, called the primary portion), and is converted into an idler that is another spectral component of the comb—hereafter called the synthesized portion (*sDKS*)—through application of the synthesis pump *sp*. This process must respect the fundamental criteria of energy and momentum conservation, which in a ring resonator translate to frequency matching and azimuthal mode number matching *ω*_SDKS_ = *ω*_pDKS_ ± ∣*ω*_PP_ − *ω*_sp_∣ and *μ*_SDKS_ = *μ*_pDKS_ ± ∣*μ*_PP_ − *μ*_sp_∣, respectively. Using the integrated dispersion previously defined, these fundamental conditions can be summarized in a single equation (see Supplementary Material Section I).

1 $$({\mu }_{{{{{{{{\rm{pDKS}}}}}}}}}\pm {\mu }_{{{{{{{{\rm{pp}}}}}}}}}){D}_{1} =	 \,({D}_{{{{{{{{\rm{int}}}}}}}}}({\mu }_{{{{{{{{\rm{sDKS}}}}}}}}}^{\pm })\pm {D}_{{{{{{{{\rm{int}}}}}}}}}({\mu }_{{{{{{{{\rm{sp}}}}}}}}}))\\ 	+({\mu }_{{{{{{{{\rm{sp}}}}}}}}}\pm {\mu }_{{{{{{{{\rm{sDKS}}}}}}}}}^{\pm }){D}_{1}$$

This results in a simple pair of conditions for FWM-BS based on the idler that is considered:

2 $$\left\{\begin{array}{l}{{\Delta }}{D}_{{{{{{{{\rm{int}}}}}}}}}^{-}={D}_{{{{{{{{\rm{int}}}}}}}}}({\mu }_{{{{{{{{\rm{sDKS}}}}}}}}}^{-})-{D}_{{{{{{{{\rm{int}}}}}}}}}({\mu }_{{{{{{{{\rm{sp}}}}}}}}})=0\\ {{\Delta }}{\mu }^{-}={\mu }_{{{{{{{{\rm{sDKS}}}}}}}}}^{-}-{\mu }_{{{{{{{{\rm{sp}}}}}}}}}={\mu }_{{{{{{{{\rm{pDKS}}}}}}}}}-{\mu }_{{{{{{{{\rm{pp}}}}}}}}}\end{array}\right.$$

3 $$\left\{\begin{array}{l}{{\Delta }}{D}_{{{{{{{{\rm{int}}}}}}}}}^{+}={D}_{{{{{{{{\rm{int}}}}}}}}}({\mu }_{{{{{{{{\rm{sDKS}}}}}}}}}^{+})+{D}_{{{{{{{{\rm{int}}}}}}}}}({\mu }_{{{{{{{{\rm{sp}}}}}}}}})=0\\ {{\Delta }}{\mu }^{+}={\mu }_{{{{{{{{\rm{sDKS}}}}}}}}}^{+}-{\mu }_{{{{{{{{\rm{pp}}}}}}}}}={\mu }_{{{{{{{{\rm{pDKS}}}}}}}}}-{\mu }_{{{{{{{{\rm{sp}}}}}}}}}\end{array}\right.$$

Here the superscript ± denotes the two kinds of idler that can result from a FWM-BS process, one at higher frequency (+) and the other at lower frequency (−) than the signal. Due to phase-matching considerations, only one of each would match the FWM-BS condition for a given signal.

The first case to consider is for a signal that is close to the main pump (Fig. 1a). Only the low frequency idler satisfies the condition in Eq. (2). Therefore, the FWM-BS process translates a DKS comb tooth close to the primary pump into a comb tooth close to the synthesis pump. As the mismatch in the integrated dispersion ${{\Delta }}{D}_{{{{{{{{\rm{int}}}}}}}}}^{-}$ (i.e. mismatch in the fundamental energy conservation of the FWM process) increases with the mode spacing Δ*μ*^−^, the efficiency of the FWM-BS process decreases, giving rise to the Lorentzian spectral shape around the synthesis pump. However, comb teeth close to the primary pump are not the only ones that can be efficiently spectrally translated. Due to the higher order dispersion coefficients leading to a roll-off of the integrated dispersion, the energy conservation condition ${{\Delta }}{D}_{{{{{{{{\rm{int}}}}}}}}}^{-}=0$ is met again for large enough mode spacing (Fig. 1b). Therefore, the mode frequency exhibiting the same integrated dispersion value as at the synthesis pump would exhibit efficient FWM-BS. This process extends the frequency comb bandwidth on the low frequency side, with a new DW generated near 160 THz. The system we consider presents a large asymmetry in the integrated dispersion, and therefore the condition ${{\Delta }}{D}_{{{{{{{{\rm{int}}}}}}}}}^{-}=0$ is only met once. However, even for symmetric integrated dispersion in which there is an additional ${{\Delta }}{D}_{{{{{{{{\rm{int}}}}}}}}}^{-}=0$ on the high frequency side, no corresponding high frequency DW will be generated through this negative idler process. This is because the phase-matching criterion from Eq. (2) requires the mode spacing between the synthesis pump and the synthesized portion to match the separation between the primary pump and the primary portion, and such a separation is too large for the primary portion to contribute an adequate power signal for the FWM-BS process.

That being said, Eq. (3) indicates that a higher frequency idler can be generated for any modes whose integrated dispersion is equal and opposite in sign to the integrated dispersion value at the synthesis pump. A comb tooth from the synthesis portion ${\mu }_{{{{{{{{\rm{sDKS}}}}}}}}}^{+}$ must respect momentum conservation, which in this case means that the mode spacing between the signal (comb tooth from the primary portion) and the synthesis pump must match the mode spacing between the idler (comb tooth from the synthesized portion) and the primary pump. Once again, due to the higher order dispersion that allows a zero crossing of the integrated dispersion, negative values of *D*_int_ are possible, and an efficient FWM-BS process can happen at frequencies beyond the original high-frequency DW of the single-pumped DKS. This results in a new DW near 409 THz, extending the bandwidth of the frequency comb to higher frequencies, i.e. toward the visible. Interestingly, another frequency matching condition in our case would occur at low frequency (near 138 THz), as another zero crossing of the integrated dispersion happens on this side of the spectrum. However, momentum conservation is not respected, as the idler must have a higher mode number than the signal, and therefore no FWM-BS happens here.

Using this fundamental property that FWM-BS translates the primary DKS comb teeth into new spectral regions while maintaining the comb tooth spacing, we introduce *D*_synt_ (Fig. 1d), a synthetic dispersion that captures the essence of the FWM processes we have presented. *D*_synt_ is an effective integrated dispersion that incorporates the combined influence of the geometric integrated dispersion and the synthesis-pump-induced FWM-BS processes. *D*_synt_ is essentially a piece-wise shifted version of *D*_int_, with the two being equal in the spectral region surrounding the primary pump, and differing in the regions where FWM-BS causes a broadening of the spectrum and the generation of new DWs. In these regions, we simply use the FWM-BS conditions for creating the new DWs to determine how to shift *D*_int_ (either up or down) so that its zero crossings are appropriately located. Stitching together the different regions of the synthetic dispersion is accomplished by taking into account FWM-BS power considerations, and in particular, where FWM-BS is less efficient. In our case, this is at the midpoint between the primary pump and the synthetis pump, and in between the primary DW (i.e., that generated by the original DKS) and the high frequency FWM-BS DW. This approach provides a linear approximation of where the DWs will be created (*D*_synt_ = 0) and helps estimate the spectral extent of the frequency comb. To test its validity, we simulate the behavior of the system using a generalized version of the Lugiato–Lefever Equation (LLE), described in detail in the Supplementary Material Section III. This version of the equation has not been subjected to typical simplifications, and in particular, the pumped modes remain as phase terms relative to the center of the frequency domain, so that the evolution of the intracavity electric field under multiple driving fields can be studied^25^.

### Ultra-broadband microcombs

**Fig. 2:**
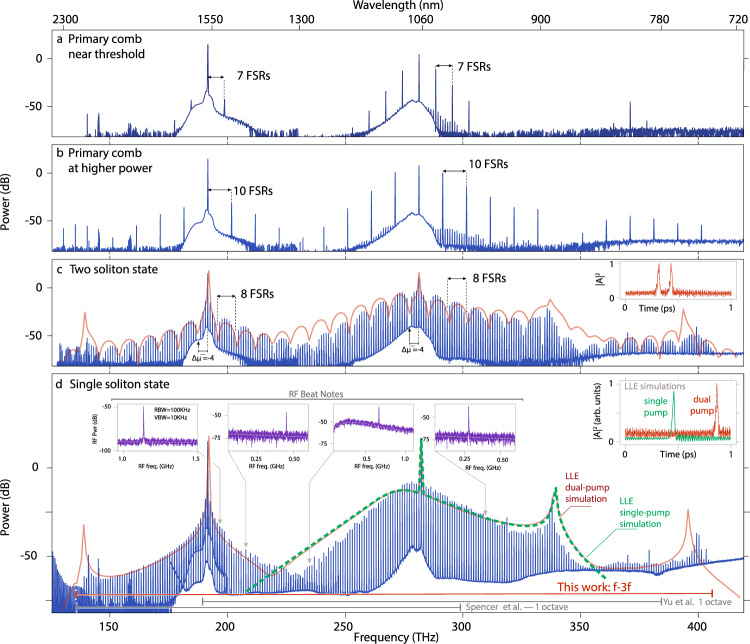
Spectral translation to create ultra-broadband microcombs. A microring resonator with *RW* = 1117 nm is pumped by a primary pump at 282 THz and synthesis pump at 192 THz. **a** Primary comb generation with low primary pump power near threshold. The comb spacing is equal to seven free spectral ranges (FSRs) and reproduced around the synthesis pump and idlers, highlighting the mixing process between the two pumps and the primary portion comb teeth. **b** Primary comb generation at a higher primary pump power where, as previously, the spectral spacing in the primary portion is matched by that in the synthesis portion, as expected by the FWM-BS theory. **c** Two-soliton state, where the characteristic 8 FSR modulation in the comb envelope is replicated near the synthesis pump. The inset shows the LLE-calculated two-soliton pulse arrangement that results in the simulated comb envelope shown in red. We highlight the missing comb tooth in the primary portion (Δ*μ* = −4), whose absence is translated onto the synthesized portion of the comb, respecting the FWM-BS phase-matching condition. **d** Single soliton state, where the impact of the synthesis pump is to expand the comb bandwidth to 1.6 octaves and create new DWs on both ends of the spectrum. The spectrum agrees with the generalized LLE solution using the dual-pump model (red line), and greatly exceeds the expected spectrum if just the primary pump is applied (dashed green line). The phase-coherent nature of the comb is verified through beat note measurements with narrow linewidth lasers throughout the comb spectrum (four left insets). The noise floor for each measurement is shown in dashed lines, and is higher in the O-band due to use of an additional RF amplifier. The rightmost inset shows the LLE simulation of the expected time-domain behavior under dual pumping (red) and if only the primary pump is applied (green). The horizontal bars at the bottom of the graph compare the span achieved here with octave-spanning DKSs from refs. ^3, 32^. We note that the low frequency portion of the spectrum exhibits OSA artefacts, at 146, 159, and <135 THz; the shortest DW at 141 THz is not impacted by these artefacts. 0 dB is referenced to 1 mW, i.e., dBm.

To experimentally study the above phenomena, we perform measurements on Si_3_N_4_ microring resonators whose design and basic characterization are described in the Methods and Supplementary Material. The resonators are pumped in two bands, with a primary pump around 1063 nm and a synthesis pump around 1557 nm, and the coupling enabled by a tailored pulley waveguide geometry that realizes a relatively flat coupling rate across a wide spectral range^31^ (see Supplementary Material Section V). We first show the spectral translation nature of the dual-pump system for different microcomb states. Figure 2a, b shows the spectral behavior for primary combs generated just above threshold and at a higher power, respectively, where in both cases we observe that the comb tooth spacing (7 FSRs and 10 FSRs, respectively) surrounding the primary pump is retained around the synthesis pump and the higher frequency region between 350 to 400 THz. This behavior persists as we reach the soliton regime, in which the synthesis pump provides both a new nonlinear mixing mechanism (FWM-BS) as well as thermal stabilization, with clear signatures of soliton steps observed (Supplementary Material Fig. S3). Figure 2c shows the results for a two-soliton state, and in Fig. 2d, spectral translation of a single soliton state and the generation of additional DWs that greatly expand the comb spectrum is demonstrated. In each of these states, the spectral separation between the comb lines remains the same between the primary portion and the synthesized one, illustrating that the comb lines from the synthesized portion are unlikely to be due to the synthesis pump alone, and instead are a result of the mixing between both pumps and the primary portion comb lines. In addition, the clear translation of the two-soliton comb envelope modulation pattern onto the synthesis component confirms this point. This spectral pattern (due to the relative phase of the two pulses circulating in the cavity) effectively acts as a modulation of the signal power in the FWM-BS process, and the replication of this pattern in the synthesized portion of the comb follows the expectation for FWM-BS that the generated idler power is linearly proportional to the input signal power. For example, a comb tooth 4 FSRs below the primary pump (Δ*μ* = − 4) is absent (likely due to an avoided mode crossing), and this is replicated by the absence of a comb tooth 4 FSRs below the synthesis pump. This also emphasizes the phase-matching condition and is consistent with the FWM-BS framework described previously.

**Fig. 3:**
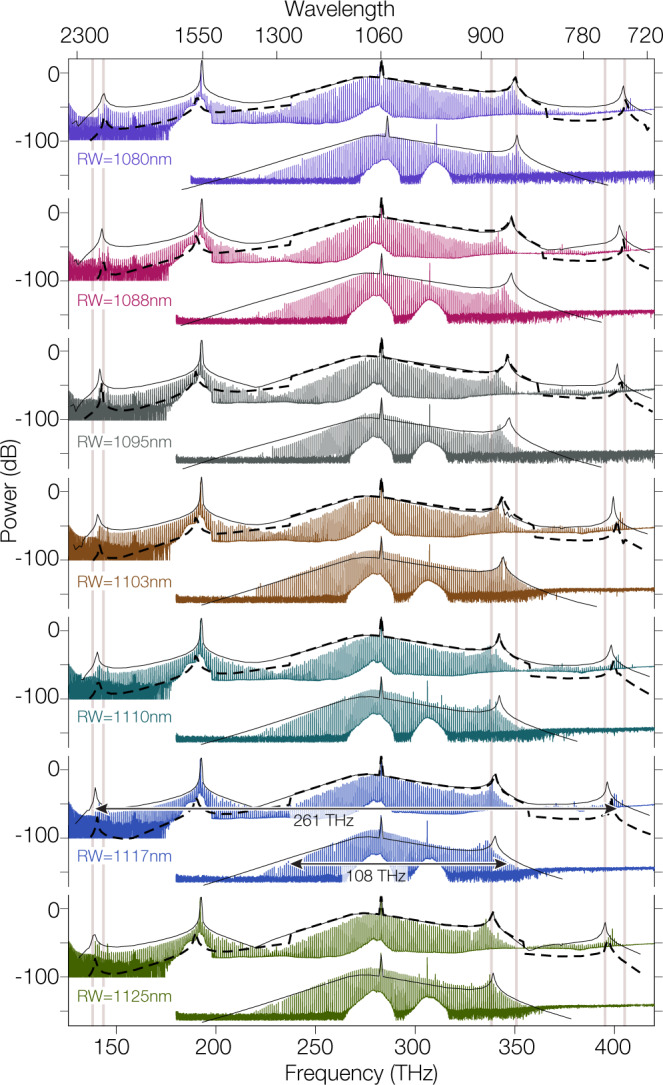
Ultra-broadband soliton spectral translation: geometric dependence. Geometric dispersion impacts the location of the generated DWs, much like the case in singly pumped DKS devices. Here, microrings are pumped at 192 and 282 THz with a pump power of 200 and 250 mW respectively, for ring widths (RWs) from 1080 to 1125 nm. In each case, an ultra-broadband microcomb is generated in which the soliton comb teeth surrounding the primary pump are spectrally translated by the synthesis pump. Single-pump DKS states are also showcased for each RW, where thermal stability was obtained through cross-polarized counterclockwise pumping at 305 THz. The solid black lines represent the expected single soliton spectra calculated through the dual-pumped generalized LLE and single-pump LLE for their corresponding counterpart experiments. The dashed lines represent the microcomb envelope prediction using the synthetic dispersion and single-pump LLE. The light gray solid lines are visual guides showcasing the shift of the DWs with RW. 0 dB is referenced to 1 mW, i.e., dBm.

When reaching the single soliton state, the comb extends from 137 to 407 THz, a span allowing f-3f stabilization and a significant increase in bandwidth relative to state-of-the-art DKS microcombs^3,32^. The comb envelope is in good agreement with the predictions of the generalized LLE model, which incorporates both the primary and synthesis pumps, in terms of the overall comb envelope, the spectral positions of the different DWs, and the >80 dB dynamic range in comb tooth power across the ultra-broadband spectral range. Finally, we note that the LLE provides insight about the nature of the intracavity field (Fig. 2c, d, insets). In the time domain, it predicts a two-soliton pulse (Fig. 2c) and a single soliton pulse (Fig. 2d) with an estimated pulse duration (full-width at half-maximum) of 16 fs. The pulses sit on a modulated background characteristic of DWs on both sides of the spectrum, and the pulse itself shows some amount of structure. In contrast, we also plot the expected time-domain behavior for a singly pumped soliton state with a single DW (Fig. 3d), where the background modulation is larger on one side of the pulse, and the pulse itself shows no additional structure. This highlights the continuous wave nature of the translated portion of the spectrum, which does not create new pulses, but rather increases the background modulation in the same manner as DWs do. We note that the pulsed behavior from the time-domain simulations is consistent with coherence across the whole 1.6 octave bandwidth of the comb.

To probe the comb coherence in the single soliton case, we perform beat note measurements with narrow linewidth tunable lasers (at 970, 1270, 1420, and 1520 nm) positioned at different locations within the comb spectrum, covering many different parts of the comb with different spectral shapes. In each case (inset to Fig. 2c), the beat note is a single tone, which is a signature of the phase-coherent nature of the frequency comb^33,34^ and is to be contrasted with the multiple beat notes that might be expected for modulation instability processes that lead to sub-comb formation. The beat notes at 1270 and 970 nm evaluate the primary portion of the DKS, and are thus expected to exhibit clear single tones, reflecting the single pulse nature of the DKS in the resonator. The beat notes at 1420 nm and at 1520 nm, which lie in spectrally translated portion near the synthesis pump, support the hypothesis of the binding of the synthesis pump with the single soliton and the coherence of the synthesized component of the comb. Later, we will strengthen these conclusions through heterodyne measurements between the two portions of the comb.

### Soliton spectral translation: geometric dependence

To explicitly demonstrate the impact of the synthesis laser on spectral translation of soliton microcombs, in Fig. 3 we compare spectra generated when both primary and synthesis pump lasers drive nonlinear processes in a series of microresonators to the case where only the primary pump drives soliton generation. Thermal stabilization in the latter case is achieved by a counterpropagating cross-polarized laser at 980 nm; the opposite propagation direction and orthogonal polarization ensure that its impact on nonlinear dynamics is minimized. For each microring, we see that effect of the synthesis laser is to mediate spectral translation and new DW generation, while leaving the portion surrounding the original soliton state essentially unchanged, as predicted from Fig. 1. The microrings differ only in their ring widths, which range from 1080 to 1125 nm, and since the microring cross-section strongly influences the integrated geometric dispersion and the resulting synthetic dispersion under dual pumping, we expect this *RW* variation to impact the generated comb spectra and the DW positions. Each device exhibits an ultra-broadband spectrum, and as expected, all DWs tune with ring width. The generalized LLE described in the Supplementary Material Section III provides good agreement (solid black lines in Fig. 3) with the obtained experimental spectra and reproduces the observed DW tuning. In addition, the single-pumped synthetic dispersion simulations (dashed black lines in Fig. 3) provide similar predictions for the comb envelope and DW positions, highlighting the utility of our heuristic model and its use as a predictive tool to design ultra-broadband frequency combs. However, it is important to note that both the single-pump LLE and the more generalized *N*-pump LLE rely on the basic assumption of a single fixed frequency grid, indexed by the mode number *μ*, through Fourier transform of the fast time temporal profile. Hence these models do not capture any frequency discrepancy between the DKS teeth and the nearest cavity resonances.

### Composite frequency comb overlap frequency offset

**Fig. 4:**
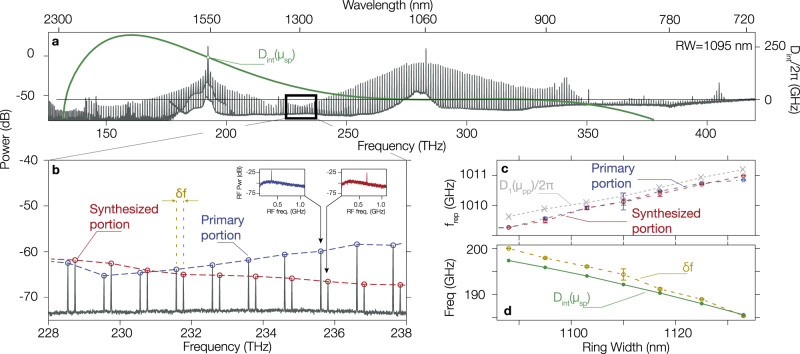
Overlap of the primary and synthesized portions of the comb spectrum. **a** Spectrally translated single DKS spectrum with the highlighted overlap region. The integrated dispersion computed at the primary pump frequency (282 THz) is shown in green, with an annotation highlighting the expected frequency offset between the DKS primary portion comb tooth and the cavity resonance at the synthesis pump frequency. **b** Zoom-in of the overlap region, highlighting the primary portion of the DKS (blue) and the synthesized portion (red). The beat note of pairs of adjacent comb teeth separated by the overlap-offset frequency *δf* are displayed in the insets. **c** Measured comb tooth spacing for both the primary and synthesized portion in the overlap region, exhibiting a uniform value across the spectrum that is ring width dependent. The linear FSR around the main pump is measured and reported in the gray dashed line, and is close to the measured repetition rate of the DKS. The error bars represent the variance of the measured *f*_rep_ for different pairs of comb teeth. **d** Measurement of the overlap-offset frequency *δf* and the predicted value of the integrated dispersion about the primary pump, evaluated at the synthesis pump frequency (*D*_int_(*μ*_sp_)). The overlap-offset frequency is uniform across the overlap region, and is well-predicted by *D*_int_(*μ*_sp_), indicative of the intrinsic detuning between the DKS teeth and the cavity resonances frequencies. The error bars represent the variance of the measured *δf* for different pairs of comb teeth. 0 dB is referenced to 1 mW, i.e., dBm.

Closer inspection of Fig. 2d and every spectrum in Fig. 3 reveals an important feature: the primary portion and the synthesized portion do not overlap perfectly, resulting in a composite frequency comb. Although we are pumping the same mode family in both bands, the overlap region in the comb spectrum (Fig. 4a, b) exhibits pairs of adjacent comb teeth with an overlap-offset frequency *δf* = *f*_sDKS_ − *f*_pDKS_ that is smaller than the repetition rate of the DKS. We now consider whether *δf* remains the same across this ‘stitching’ region.

We characterize the comb tooth spacing, i.e. the repetition rate *f*_rep_ in the primary and synthesized portions of the comb, for the state shown in Fig. 4a. This is done by measuring beat notes between a helper laser and the comb teeth, with the helper laser wavelength determined by a wavemeter (accuracy ≈ 50 MHz). Through measurement across the helper laser tuning range (228 THz to 238 THz), we find that (Fig. 4b) *f*_rep_ is uniform and equal for both the primary and synthesized portions, and its value is close to the FSR around the primary pump (equivalently, *δf* is uniform throughout the overlap region). We then repeat measurement of *f*_rep_ and *δf* for devices with different *RW*, and find that these conclusions hold, with the specific measured values dependent on *RW* (Fig. 4c, d). These conclusions are understood by the fact that the FWM-BS process must respect frequency matching, and thus the soliton repetition rate will be transferred to the synthesized portion of the comb.

In addition, contrary to the strong coupling in a dual-pump system that has been theoretically studied^25,35^, the synthesis pump here does not change the DKS repetition rate or shift the frequency of resonance enough to enter the regime where Arnold tongues and sychronization might exist^36^. We believe that such locking cannot happen in our devices because the overlap frequency shift cannot be compensated by the Kerr shift at the synthesis pump (≈−1.3 GHz). Thus, this overlap-offset frequency between the primary and synthesized portions of the comb can be understood as the fundamental discrepancy between the soliton comb tooth frequency and the cavity resonance frequency^37^. As such, we expect that *δf* should be given by the value of the primary pump integrated dispersion evaluated at the synthesis pump frequency *D*_int_(*μ*_*sp*_), in the absence of strong overlap between the primary and the synthesized elements, which is our case since the synthesis pump is placed outside of the primary soliton spectral envelope. In Fig. 4d, we compare *δf* and the theoretical value of *D*_int_(μ_sp_), and find that they are in good agreement and within the expected Kerr shift (couple of GHz) induced by the synthesis pump.

### Probing the relative noise between the two portions of the comb

**Fig. 5:**
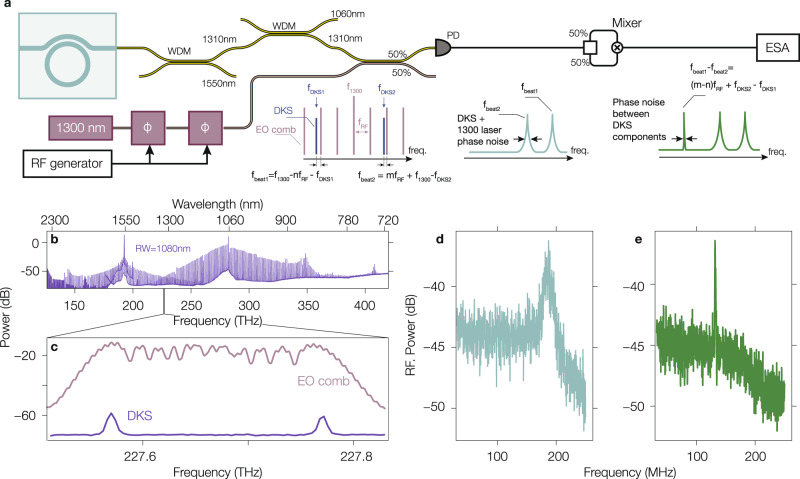
Relative phase noise between the primary and synthesis portions. **a** Schematic of the measurement setup through which the relative phase noise of two comb teeth in the stitching region, one from the primary comb portion and one from the synthesis comb portion, is measured with the assistance of a 1300 nm EO comb. **b** Spectrum of the microcomb device under investigation. **c** Two microcomb teeth from the stitching region (blue) plotted along with the EO comb (purple). The EO comb teeth are not fully resolved due to the limited spectral resolution of the optical spectrum analyzer in comparison to the EO comb repetition frequency (6.0156 GHz). **d** Beat note of the EO comb with the two microcomb teeth. Only one out of the two beatnotes is present due to the limited spectrum analyser bandwidth (250 MHz). **e** Mixing of the two beat notes produced between the EO comb and the microcomb teeth to retrieve the relative phase noise of the microcomb teeth, without the (free-running) 1300 nm laser phase noise contribution. Both spectra have been taken with a resolution bandwidth of 230 kHz.

Thus far we have shown that the spectrum produced by our dual-pump system is consistent with the picture in which FWM-BS mediates soliton spectral translation, with new DWs generated on both the low and high frequency sides of the spectrum. Moreover, we have explicitly shown that the comb tooth spacing is translated, and there is an overall shift between the two portions of the spectrum. In addition, the narrow heterodyne beat notes across the spectrum suggest that the primary and synthesis portions of the comb each exhibit low noise, but so far, their relative noise has not been experimentally considered. Now, if FWM-BS is indeed the dominant process in the spectral broadening of the primary DKS state into an ultra-broadband comb, we should expect that no added phase noise (other than that of the synthesis laser) will be accrued on the synthesis component teeth, and that there will be low noise between the synthesis portion and the primary portion of the comb. To investigate this, we perform a coherent heterodyne measurement of the two portions of the comb to extract their relative phase noise. Because of the large frequency offset *δf* ≈ 200 GHz, as observed earlier, we are unable to directly measure such a large beat frequency. Instead, we effectively mix the two comb teeth of interest using an intermediate electro-optic (EO) comb whose driving laser phase noise is suppressed through the heterodyne setup. The EO comb is realized by modulating a 1300 nm laser with two EO phase modulators at a repetition rate of 6.0156 GHz and with an overall bandwidth that spans across the frequency gap *δf* between a single comb tooth of the primary and secondary portions (Fig. 5a–c). As the 1300 nm laser that seeds the EO comb is free-running and not locked to the microcomb (which itself is free-running), the obtained individual beat notes between each DKS comb tooth and the EO comb are relatively broad (Fig. 5d), where only one beatnote is observed since their separation is larger than the spectrum analyzer bandwidth (250 MHz). However, by mixing together the two beat notes in a specific configuration, that is, so that the DKS comb teeth are on the same side of the EO comb teeth for both the primary and secondary portions of the microcomb, we are able to suppress the 1300 nm seed laser phase noise (Fig. 5a, e). This approach effectively downconverts, by a fixed factor determined by the EO comb repetition rate and its comb teeth used to realized the beating with the DKS, the overlap-offset frequency to a frequency which can be easily measured without the introduction of additional noise (assuming the RF generator driving the EO comb has negligible noise). The expected behavior based on an underlying nonlinear process dominated by the FWM-BS is corroborated in experiment, where a narrow single tone, indicating a low-noise state between the two portions of the comb, is observed (Fig. 5e). Given that a DKS soliton (the primary portion of the comb) is a low-noise state, this suggests that the spectral translation process has resulted in a synthesis portion that is also low-noise. Though stabilization of the DKS and the pump lasers has not been performed, limiting the ability to draw a final conclusion about the phase coherence between the two portions of the comb, considering the earlier measurements and theoretical analysis, which show that the repetition rate in both portions of the comb is unvarying and identical to within our measurement capability, suggests—as expected from the physical picture of FWM-BS—that the soliton spectral translation process results in a single, fully phase-coherent comb. The frequency of each comb tooth is then known once there is knowledge of the repetition rate, the carrier-envelope offset frequency, and the overlap-offset frequency *δf*, whose value is determined by the integrated dispersion value at the synthesis pump.

Hence, based on the experimental demonstrations throughout this work, it seems likely that the ultra-broadband microcombs we have demonstrated can be used for metrological purposes, such as *f* − 2*f* self-referencing, provided that, along with the comb repetition frequency and carrier-envelope offset frequency, the overlap-offset frequency *δf* is measured. In such a scenario, where both pumps can be independently stabilized, the large power available from the synthesis pump would be of particular appeal, as it could be efficiently frequency doubled and, through proper dispersion engineering of the resonator, could be made resonant with the high frequency DW.

## Discussion

Zhang and colleagues have recently realized a similar dual pumping configuration, in which an auxiliary laser at 1330 nm spectrally broadens a 1550 nm soliton microcomb down to wavelengths of around 1275 nm^30^, considerably increasing the bandwidth of such low repetition rate DKS solitons. The spectrum extends from 1275 to 1720 nm (taken at the −50 dB points relative to the maximum), an extent of 60 THz, and the auxiliary laser is responsible for about 22 THz of spectral extension on the high frequency side, resulting in about a factor 1.5 × increase of the bandwidth of the comb. The work presented in this manuscript provides significant new insight in the physical mechanism behind these new effective DWs, and the possibility to extend the microcomb bandwidth significantly on both sides of the spectrum. Indeed, here our 1557 nm auxiliary laser causes a broadening of the comb spectrum on both low and high frequency sides of the original soliton spectrum centered at 1063 nm, with a comb extending from 737 to 2190 nm, an extent of 270 THz, and the auxiliary laser is responsible for about 93 and 79 THz of spectral extension on the low and high frequency sides, respectively (173 THz broadening in total), in our case extending the comb by more than a factor 2.6. Perhaps more important than the characteristics of the comb broadening is its fundamental physical origin. In ref. ^30^, spectral broadening is generally attributed to XPM effects, which leaves open many questions about the relationship between the original comb and the spectrally broadened region—in particular, whether any offset between the two regions is indicative of independent frequency combs. Here, we show that the dominant process behind spectral broadening in our system is FWM-BS, so that soliton comb teeth generated by the primary pump are spectrally translated to both the low frequency and high frequency sides of the spectrum. In addition, we show that the overlap-offset frequency between the primary region of the comb and the spectrally translated one is inherent to the microring resonator geometry, and is not a signature of two independent frequency combs. In particular, the natural discrepancy between the DKS comb teeth and cavity resonance frequency causes the synthesis pump to be offset from the nearest DKS comb tooth by the value of the integrated dispersion at this pumped mode, which we confirm through measurement of the overlap-offset frequency as a function of ring geometry. Finally, we have performed several measurements that indicate that the FWM-BS process in our system directly translates primary DKS comb teeth to another spectral window, resulting in a comb state with low-noise in each portion as well as in the overlap region, pointing to the potential use of such ultra-broadband frequency combs for metrological purposes. This allows us to introduce a simplifying tool for designing this new kind of frequency comb, by summarizing the nonlinear interaction and the position of all the DWs generated by the dual-pump system through the synthetic dispersion. Such coherent ultra-broadband frequency combs through DKS spectral translation could find many applications, in particular, by harnessing the high power of the synthesis pump, which would aid in *f*-2*f* self-referencing, for monolithic integration of *f*-3*f* within a *χ*^(3)^ platform, and by pushing the limit of coherent DW generation further into the visible.

## Methods

### Device design

We use 775 nm thick Si_3_N_4_ ring resonators, which were fabricated at Ligentec SA, with a fixed ring radius of 23 μm, a ring width (*RW*) that is varied between 1088 and 1140 nm across the devices, and a surrounding silica cladding. The access waveguides for coupling to/from the rings are tapered down to 200 nm at the facets, resulting in about 6 and 5 dB insertion losses per facet at 192 and 282 THz respectively. We use a pulley waveguide with a width of *W* = 550 nm, a length of *L*_c_ = 9 μm, and a gap *G* = 370 nm. The expected frequency-dependent coupling, computed using the coupled mode theory formalism developed in ref. ^31^, exhibits a resonance-free spectrum and *Q*_*c*_ that varies within one order of magnitude over an octave (Supplementary Material Fig. S2).

## Supplementary information

Supplementary Information

## Data Availability

The data that supports the plots within this paper and other findings of this study are available from the corresponding authors upon reasonable request.
