# Supplementary material for: Ultra-broadband Kerr microcomb through soliton spectral translation
Source: Nat Commun. 2021 Dec 14;12:7275. doi: 10.1038/s41467-021-27469-0 (PMC8671399; doi:10.1038/s41467-021-27469-0)
Supplement: Supplementary file 1 — Supplementary Information [file 41467_2021_27469_MOESM1_ESM.pdf]

Supplementary Materials – Ultra-Broadband Kerr Microcomb Through Soliton Spectral Translation

Gregory Moille,[1,2,*] Edgar F. Perez,[1,2] Jordan R. Stone,[1,2] Ashutosh Rao,[2,3] Xiyuan Lu,[2,3] Tahmid Sami Rahman,[1] Yanne K. Chembo,[3] and Kartik Srinivasan[1,2,†]

[1] *Joint Quantum Institute, NIST/University of Maryland, College Park, MD, USA*
[2] *Microsystems and Nanotechnology Division, National Institute of Standards and Technology, Gaithersburg, MD, USA*
[3] *Institute for Research in Electronics and Applied Physics, University of Maryland, College Park, MD, USA*

I. FOUR-WAVE MIXING BRAGG SCATTERING MATCHING CONDITIONS

Four-wave mixing (FWM) relies on the basic conditions of energy and momentum conservation, which in a microring resonator translate to frequency (ω) and azimuthal mode number (M) conservation. In the case of FWM Bragg Scattering (FWM-BS), the signal photon (which in our case is a comb tooth from the primary portion of the comb, labeled $pDKS$) can be scattered to two spectrally-translated idlers, labeled as $sDKS^{\pm}$, such that these fundamental energy/momentum conservation equations become:

$$\begin{cases} \omega_{\mathrm{pDKS}} \pm \omega_{\mathrm{pp}} = \omega^{\pm}_{\mathrm{sDKS}} \pm \omega_{\mathrm{sp}} \\ M_{\mathrm{pDKS}} \pm M_{\mathrm{pp}} = M^{\pm}_{\mathrm{sDKS}} \pm M_{\mathrm{sp}} \end{cases} \quad \text{(S.eq.1)}$$

The frequency shift in this spectral translation process is set by the difference in the frequencies of the primary pump pp and synthesis pump sp, and both a higher frequency idler ($\omega^{+}_{\mathrm{sDKS}}$) and a lower frequency idler ($\omega^{-}_{\mathrm{sDKS}}$) relative to the signal can be generated, if they are phase- and frequency-matched [1].

In a multi-mode resonator, such as the ring resonators studied here, the integrated dispersion (D_{int}) effectively characterizes the discrepancy between the modal frequencies and that of a zero-dispersion resonator, *i.e.*, one in which all resonances are separated by the same free spectral range (FSR). D_{int} is usually referenced at the pumped mode, so that we introduce the relative mode number with respect to the pumped mode $\mu = M - M_{\mathrm{pp}}$ (*i.e* $\mu_{\mathrm{pp}} = 0$), with:

$$D_{\mathrm{int}}(\mu) = \omega_{\mathrm{res}}(\mu) - (\omega_{\mathrm{pp}} + D_1\mu) \quad \text{(S.eq.2)}$$

where $\omega_{\mathrm{res}}(\mu)$ are the cavity resonance frequencies, and $D_1 = 2\pi FSR$, computed at the primary pumped mode.

From the the above equations, we can compute the frequency of each resonance of interest from the integrated dispersion, assuming that the signal μ_{pDKS} is part of the primary DKS and that the idler $\mu^{\pm}_{\mathrm{sDKS}}$ is part of the spectrally-translated (synthesized) portion of the DKS, and hence on a fixed frequency grid relative to the synthesis pump. We then have:

$$\begin{aligned} \omega_{\mathrm{pDKS}} &= \omega_{\mathrm{pp}} + \mu_{\mathrm{pDKS}} D_1 \\ \omega_{\mathrm{sp}} &= \omega_{\mathrm{pp}} + \mu_{\mathrm{sp}} D_1 + D_{\mathrm{int}}(\mu_{\mathrm{sp}}) \\ \omega^{\pm}_{\mathrm{sDKS}} &= \omega_{\mathrm{pp}} + \mu^{\pm}_{\mathrm{sDKS}} D_1 + D_{\mathrm{int}}(\mu^{\pm}_{\mathrm{sDKS}}) \end{aligned} \quad \text{(S.eq.3)}$$

This allows us to rewrite the energy/momentum conservation condition for FWM-BS of Eq. (S.eq.1) in a single equation such that:

$$\begin{aligned} (\mu_{\mathrm{pDKS}} \pm \mu_{\mathrm{pp}})\, D_1 = &\Big(D_{\mathrm{int}}(\mu^{\pm}_{\mathrm{sDKS}}) \pm D_{\mathrm{int}}(\mu_{\mathrm{sp}})\Big) + \\ &(\mu_{\mathrm{sp}} \pm \mu^{\pm}_{\mathrm{sDKS}})\, D_1 \end{aligned} \quad \text{(S.eq.4)}$$

Because of the asymmetric nature of the integrated dispersion (due to odd order dispersion coefficients), both high and low frequency idlers will be obtained for different input signals to respect the above equation. First, we study the case of the lower frequency idlers, as described in Fig 1(a-b). Equation S.eq.4 in this case becomes:

$$\begin{aligned} (\mu_{\mathrm{pDKS}} - \mu_{\mathrm{pp}})\, D_1 = &\Big(D_{\mathrm{int}}(\mu^{-}_{\mathrm{sDKS}}) - D_{\mathrm{int}}(\mu_{\mathrm{sp}})\Big) + \\ &(\mu^{-}_{\mathrm{sDKS}} - \mu_{\mathrm{sp}})\, D_1 \end{aligned} \quad \text{(S.eq.5)}$$

For efficient idler generation, we start by assuming that momentum is conserved to allow FWM-BS. In the frequency comb, this is trivial as it involves the simple condition that the number of resonator modes separating the primary pump and the signal $\mu_{\mathrm{pDKS}} - \mu_{\mathrm{pp}}$ must be the same as the number of modes separating the synthesis pump and the idler $\mu^{-}_{\mathrm{sDKS}} - \mu_{\mathrm{sp}}$. This will be respected due to the large number of comb teeth that can act as the signal. Assuming these two conditions, frequency- and phase-matching for FWM-BS becomes:

$$\begin{cases} D_{\mathrm{int}}(\mu^{-}_{\mathrm{sDKS}}) = D_{\mathrm{int}}(\mu_{\mathrm{sp}}) \\ \mu^{-}_{\mathrm{sDKS}} - \mu_{\mathrm{sp}} = \mu_{\mathrm{pDKS}} - \mu_{\mathrm{pp}} \end{cases} \quad \text{(S.eq.6)}$$

This implies that any resonator mode, as long as it is at a lower frequency than the the largest frequency comb tooth, will undergo FWM-BS if it exhibits the same integrated dispersion value as that at the synthesis pump frequency. The power of this idler will be limited by the primary comb tooth that realizes momentum conservation. In our case, we have the synthesis pump at a lower

* gmoille@umd.edu
† kartik.srinivasan@nist.gov

frequency than the primary pump, so that this condition describes both the frequency comb shape around the synthesis pump and the newly created DW at lower frequency.

On the other hand, the higher frequency idler respects the equation:

$$D_{\mathrm{int}}(\mu^{+}_{\mathrm{sDKS}}) + \left(\mu^{+}_{\mathrm{sDKS}} - \mu_{\mathrm{pp}}\right) D_1 = \\ -D_{\mathrm{int}}(\mu_{\mathrm{sp}}) + \left(\mu_{\mathrm{pDKS}} - \mu_{\mathrm{sp}}\right) D_1 \qquad \text{(S.eq.7)}$$

Momentum conservation will be respected for any spacing between the primary comb tooth and synthesis pump $\mu_{\mathrm{pDKS}} - \mu_{\mathrm{sp}}$ that matches the idler and primary pump spacing $\mu^{+}_{\mathrm{sDKS}} - \mu_{\mathrm{pp}}$, leading to the condition:

$$\begin{cases} D_{\mathrm{int}}(\mu^{+}_{\mathrm{sDKS}}) = -D_{\mathrm{int}}(\mu_{\mathrm{sp}}) \\ \mu^{+}_{\mathrm{sDKS}} - \mu_{\mathrm{pp}} = \mu_{\mathrm{pDKS}} - \mu_{\mathrm{sp}} \end{cases} \qquad \text{(S.eq.8)}$$

Therefore, any resonator mode, as long as it is at higher frequency than the the lowest frequency comb tooth, and is equal and opposite in sign to the integrated dispersion value at the synthesis pump frequency will undergo a FWM-BS process. Interestingly, a primary portion DW must occur between the primary pump and the idler, as an integrated dispersion zero-crossing must happen for the above condition to hold. In Fig. 1(d) in the main text, this DW occurs at 358 THz, for example.

II. OTHER $\chi^{(3)}$NONLINEAR PROCESSES

FWM Bragg scattering can be viewed as a special case of XPM, which is far easier to notice in the spectral domain instead of the temporal domain. Therefore, we studied our system in the frequency domain with a mode per mode model (equivalent to a CMT), rather than in the temporal domain with a coupled Lugiato-Lefever equation (LLE) model. Regardless, with two pumps, many types of mixing processes may be possible, and one may ask why FWM-BS is apparently favored. To analyze this further, we consider three different cases below, and start by writing the mode number matching (phase-matching) and integrated dispersion matching (frequency-matching) for each case.

Case 1: FWM-BS

$$\mu_{\mathrm{pp}} - \mu_{\mathrm{sp}} = \mp(\mu^{\pm}_{\mathrm{sDKS}} - \mu_{\mathrm{pDKS}})$$
$$D_{\mathrm{int}}(\mu_{\mathrm{sp}}) = \mp D_{\mathrm{int}}(\mu^{\pm}_{\mathrm{sDKS}})$$

This is the case we consider in detail in the manuscript, where we show the conditions (for our geometric dispersion profile) under which higher frequency and lower frequency idlers are generated.

Case 2: pump mixing

$$\mu_{\mathrm{pp}} + \mu_{\mathrm{sp}} = \mu^{\pm}_{\mathrm{sDKS}} + \mu_{\mathrm{pDKS}}$$
$$D_{\mathrm{int}}(\mu_{\mathrm{sp}}) = D_{\mathrm{int}}(\mu_{\mathrm{sDKS}})$$

The frequency matching equation here is similar to the negative idler case for FWM-BS, but the mode number matching condition is significantly different. Therefore, the positive idler that we observe experimentally cannot be the result of pump mixing and must be the result of FWM-BS. We note that Figure 2c presents strong evidence that FWM-BS is the dominant mechanism at hand, due to the missing primary portion comb tooth at $\Delta\mu = -4$ being replicated near the synthesis pump. Pump mixing (or degenerate four-wave mixing involving only one pump field) would be unlikely to exhibit such behavior. Thus, it appears that for our geometric dispersion, pump mixing is not a significant effect.

Case 3: pure nonlinear frequency shifts

The dual pump configuration is not necessarily limited to generation of different frequency components exclusively and can additionally cause nonlinear shifts of the resonances, due to self-phase and cross-phase modulation. In the case of the four-wave mixing processes outlined above, the nonlinear frequency shifts play a role in determining the exact extent to which the interacting cavity modes are frequency matched, but do not fundamentally change the dominant nonlinear process (FWM-BS in our case) induced by the synthesis pump, because of our resonator's geometric dispersion regime and chosen pump frequencies. One might envision that in regimes where FWM-BS is particularly inefficient (e.g, very poorly frequency matched) or not respected whatsoever (e.g., the phase matching conditions in case 1 are not satisfied), either of which could occur due to different geometric dispersion and/or different synthesis and primary pump frequencies, pure nonlinear frequency shifts may play a more pronounced role, and could, for example, fundamentally change the character of the nonlinear state supported by the microresonator.

III. LUGIATO LEFEVER MODEL

The Lugiato-Lefever Equation (LLE) can be derived from basic coupled mode theory [2, 3], which is similar to the temporal approach using the nonlinear Schrödinger equation (NLSE) with the assumption of periodic boundary conditions and a slowly varying envelope [4]. In many treatments, the detuning of the pump is simplified and introduced as a linear term in the equation, making it convenient for solving a single pump system. However, it has been demonstrated in ref. [5] that avoiding such approximation allows for solving a system under N driving forces. Instead of choosing the frequency grid of the resonant modes with an origin defined by a single pump, the grid is determined by the spectral domain under study (with the origin placed at the center of the grid). The

generalized LLE becomes:

$$\frac{\partial A(t,\tau)}{\partial t} = \left(-\frac{\alpha}{2} - i\gamma L|A|^2\right) A + i t_{\mathrm{r}} \mathrm{FT}^{-1}\left[D_{\mathrm{int}}^{0} \tilde{A}(t,\omega)\right] + \sum_{\mathrm{p}} \kappa_{\mathrm{ext}}|_{\mathrm{p}} \mathcal{F}_p \mathrm{e}^{i\sigma_{\mathrm{p}}} \quad \text{(S.eq.9)}$$

where the subscript p refers to either the synthesis pump or the primary pump ($p = \{\mathrm{pp};\mathrm{sp}\}$), the index 0 represents the center of the simulation domain and hence D_{int}^{0} is the integrated dispersion of the resonator computed at the center of the simulation domain and $\mu_0 = 0$, $\tilde{A}(t,\omega) = \mathrm{FT}\left[A(t,\tau)\right]$ is the Fourier Transform of the field, $\mathcal{F}_p = -i\sqrt{P_{\mathrm{in_p}}}$ represents the pth driving field (either the primary or synthesis pump), $\sigma_{\mathrm{p}} = \delta\omega_p t + D_{\mathrm{int}}^{0}(\mu_p)t - \mu_p\theta$, represents the phase shift of the p driving field, from which it is obvious that the part relative to the fast time (or resonator angle θ) corresponds to the spectral shift away from the center of the simulation domain, and the slow time t phase corresponds to the dispersion shift away from the uniform spectral grid and the relative detuning of the p pump from its pumped mode, $\kappa_{\mathrm{ext}}|_{\mathrm{p}}$ is the waveguide-resonator coupling rate for the p pumped mode, α is the total loss of the resonator, γ is the non-linear coefficient (here assumed constant), L is the resonator round-trip length, and t_{r} is the round trip time. We note that the synthetic dispersion model replaces D_{int}^{0} with D_{synt} and a single pump field for a symmetric domain.

IV. DISPERSIVE WAVE TUNING INDEPENDENT OF GEOMETRIC AND MATERIAL DISPERSION

In this section we further justify use of the term ‘synthetic dispersion’ to capture the net effects of the new nonlinear-wave mixing processes that occur in the dual pump system. Usually, dispersion is driven by two main components. The first is material dispersion, where the chromatic dependence is such that the dispersion becomes more normal the closer the wavelength is to the band-gap, and is a significant challenge to reaching short wavelengths in integrated frequency combs. The second is geometric dispersion, where the wavelength-dependent confinement of the light within a guided mode geometry provides a modification of the phase velocity of light different from the bulk material trend, therefore providing a counter-balance to material dispersion. Thus, for a fixed platform (*i.e.* a chosen set of photonic materials), the typical view is that the geometry must be changed in order to modify the dispersion of the resonator, and hence the resulting spectral shape of the generated DKS. In this work, we have shown that by introducing the synthesis pump, we provide a new degree of freedom in controlling the comb spectrum. Apart from the significant spectral broadening mediated by the FWM-BS processes

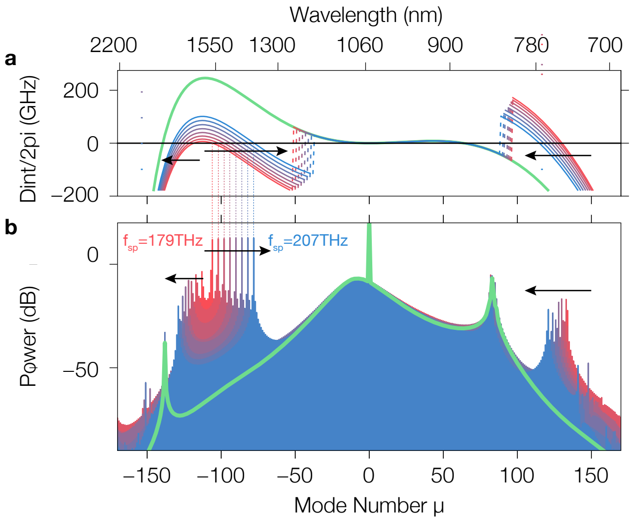

FIG. S1. | **Tuning of the newly generated DWs by tuning of the synthetic dispersion. a,** Fixed geometric integrated disperson (green) and resulting synthetic dispersion (red to blue) for different synthesis pump frequencies and a fixed primary pump frequency. **b,** Results of generalized LLE simulations showing how the comb spectrum changes as a function of synthesis pump frequency, and how the position of the newly generated DWs is predicted by the zero crossings of the synthetic dispersion curves in **a**. The solid green curve shows the spectral envelope when only the primary pump is applied.

we have described, control of the synthesis pump frequency results in tuning of the DWs (Fig. S1b). This is a consequence of a change in the synthesis pumped mode resulting in new modes that match the FWM process. The net result is that DW positions are modified without requiring modification of the geometry or material. This tuning of the DW positions with synthesis pump frequency is well-reproduced by the synthetic dispersion (Fig. S1a), whose zero-crossings closely matched the DW positions.

V. COUPLING DESIGN

In order to in-couple two pumps separated by close to 100 THz and out-couple close to a 300 THz bandwidth comb, straight waveguide coupling cannot be used as it exhibits a coupling quality factor Q_{c} (inversely proportional to the coupling rate κ_{ext}) varying by orders of magnitude over an octave [6]. However, pulley couplers exhibiting phase mismatch can achieve much more spectrally flat coupling over the bandwidth of interest. In our system, we use a pulley waveguide with a width of $W = 550$ nm, a length of $L_{\mathrm{c}} = 9$ µm, and a gap $G = 370$ nm. Such coupling, computed using the coupled mode theory (CMT) formalism developed in ref. [6], exhibits a resonance-free spectrum and Q_{c}

that varies within one order of magnitude over an octave (Fig. S2). This allows for efficient extraction of the whole ultra-broadband dual-pumped frequency comb without any gap in the spectrum, thanks to the resonance-less coupling. However, this design is quite sensitive to phase-mismatch, and as a result the geometric parameters that define the coupling, as longer pulley lengths would exhibit coupling resonances that either limit coupling of the long wavelength pump (case $L_c = 10$ µm) or the spectral region in between the primary pump and the synthesis pump would (case $L_c = 11$ µm). However, the optimal flat coupling over a large bandwidth comes at the cost that the coupling quality factor Q_c is lower than the intrinsic quality factor ($Q_i \approx 10^6$), resulting in over-coupled devices.

VI. CHARACTERIZATION SETUP

The characterization setup used in measurement of the microcomb devices is shown in Fig. S3. The resonator is pumped in the transverse electric (TE) polarization in two bands, with a primary pump at 1063 nm amplified by a ytterbium-doped fiber amplifier and a synthesis pump at 1557 nm amplified with an erbium-doped fiber amplifier, which are both coupled to the chip using a wavelength division multiplexer and a lensed fiber. The on-chip primary pump power, *i.e.* at 282 THz, is $P_{\rm pp} = 200$ mW (7.5 dB insertion loss per facet) and the on-chip synthesis pump power $P_{\rm sp} = 250$ mW (6 dB insertion loss per facet). The generated microcomb is out-coupled and split between an optical spectrum analyzer and another path allowing for beat-note measurements and comb-power detection. As expected with the synthesis pump scheme, appropriate choice of the frequency of each laser thermally stabilizes the ring resonator and results in a relatively pure Kerr response (i.e., thermal effects mitigated) with a clear signature of soliton steps for different soliton orders (inset to Fig. S3), ultimately reaching the lowest order

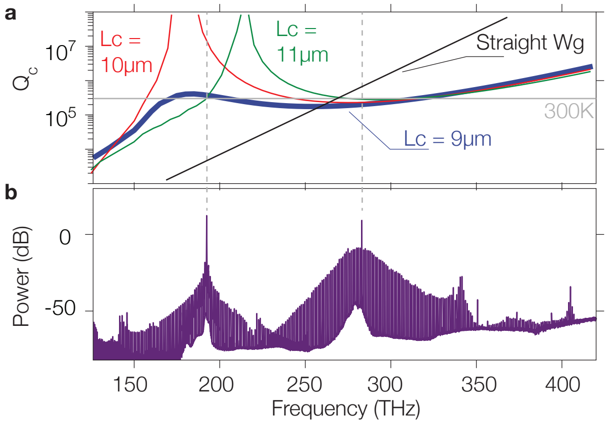

FIG. S2. | **Pulley scheme for broadband resonator-waveguide coupling**. **a,** Calculated coupling quality factor $Q_{\rm c}$ for a waveguide width = 550 nm and a ring resonator with $RR = 23$ µm and $RW = 1100$ nm, for three different pulley lengths $L_{\rm c} = \{9,\ 10,\ 11\}$ µm. $L_{\rm c} = 9$ μm provides nearly equal $Q_{\rm c}$ at the primary and synthesis pump frequencies and limited variation in $Q_{\rm c}$ across the full comb spectrum, In contrast, conventional straight coupling, shown in black, results in severe overcoupling at the synthesis pump wavelength and severe undercoupling at high frequencies. **b,** Experimental ultra-broadband DKS spectrum for $RW = 1103$ nm, illustrating the different regions of interest for in-coupling and out-coupling to/from the ring resonator. 0 dB is referenced to 1 mW, i.e., dBm.

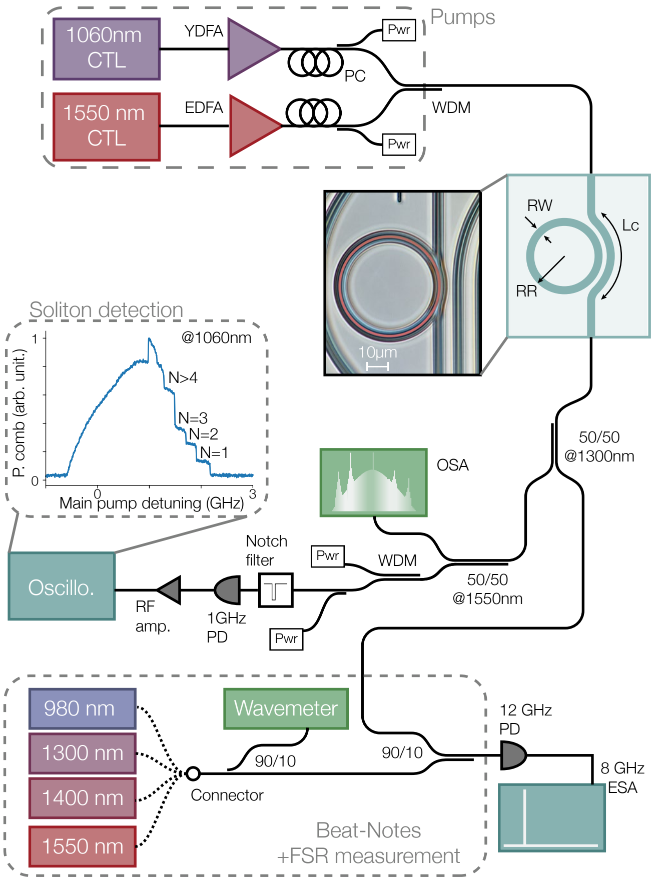

FIG. S3. | **Experimental setup.** Two amplified lasers at 282 THz (1063 nm) and 192 THz (1557 nm) pump a microring resonator. The output signal is split between different paths that allow the observation of the spectrum with an OSA and the comb power exhibiting the DKS step signature (experimental data labeled as 'Soliton detection'). One output path is used to measure the DKS comb tooth frequency through beat-note measurement with different frequency lasers and a wavemeter for absolute wavelength measurement. Pwr: Powermeter, CTL: Continuous Tunable Laser, PC: Polarization Controller, PD: PhotoDiode, OSA: Optical Spectrum Analyzer, ESA: Electrical Spectrum Analyzer, WDM: Wavelength Demultiplexer.

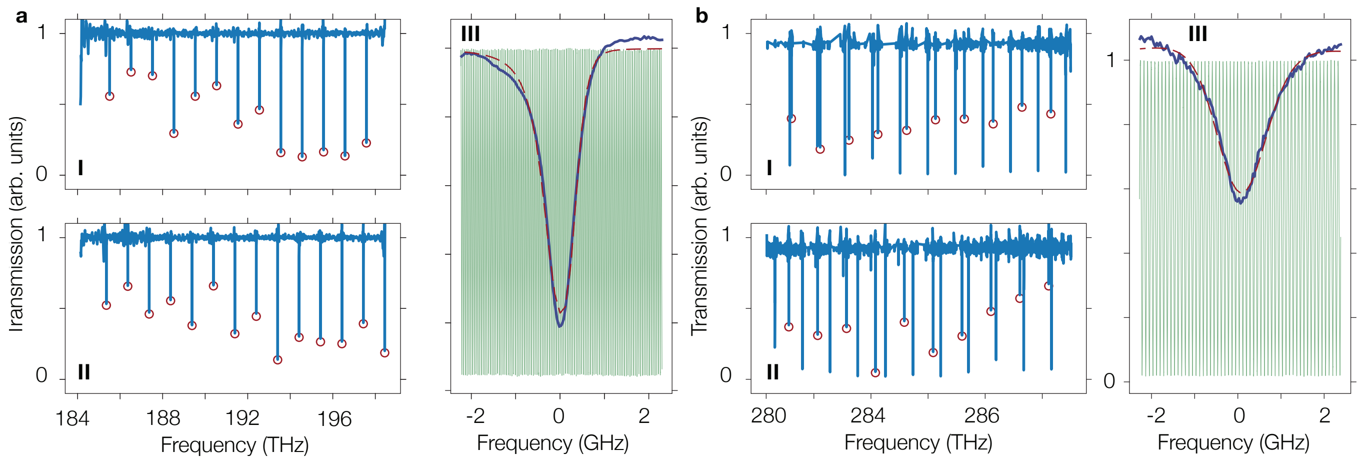

FIG. S4. | **Linear measurement of the ring resonator**. **a,** Transmission spectrum around 1550 nm for RW = 1088 nm (I) and 1103 nm (II), with a zoom-in of a representative resonance with the nonlinear least squares fit shown in the red dashed lined. The 42 MHz MZI that serves as a calibration is displayed in green. **b,** Transmission spectrum around 1050 nm for the same resonators, namely RW = 1088 (I) and 1103 nm (II), with the fit of a representative resonance. The nonlinear least squares fit to the zoomed-in data is shown as a red dashed lined in III. The 83 MHz MZI that serves as a calibration for the 1060nm band is displayed in green.

single soliton state.

VII. LINEAR MEASUREMENTS

Linear measurements to determine the mode family and the quality factor of the ring resonator, in both the 1550 and 1060 nm band, are performed by sweeping a continuous tunable laser (CTL) and recording the transmission spectrum (Fig. S4). A Mach-Zehnder Interferometer (MZI), with a free spectral range of 42 MHz and 83 MHz for the 1550 nm and 1060 nm band respectively, is used to calibrate the laser sweeps. This allows retrieval of the quality factor of the resonances in both bands, which are in the over-coupled regime as expected from the broadband-flat coupling described in the previous section. The modes in the spectra shown in Fig. S4 exhibit $Q_\mathrm{i} \approx 1.1 \times 10^6$ and $Q_\mathrm{c} \approx 3.5 \times 10^5$ for the average intrinsic and coupled quality factors, respectively.

[1] Qing Li, Marcelo Davanço, and Kartik Srinivasan, "Efficient and low-noise single-photon-level frequency conversion interfaces using silicon nanophotonics," Nature Photonics **10** (2016), 10.1038/nphoton.2016.64.

[2] T Hansson, D Modotto, and S Wabnitz, "On the numerical simulation of Kerr frequency combs using coupled mode equations," Optics Communications **312**, 134–136 (2014).

[3] Yanne K Chembo and Curtis R Menyuk, "Spatiotemporal Lugiato-Lefever formalism for Kerr-comb generation in whispering-gallery-mode resonators," Physical Review A **87** (2013), 10.1103/PhysRevA.87.053852.

[4] Stéphane Coen, Hamish G Randle, Thibaut Sylvestre, and Miro Erkintalo, "Modeling of octave-spanning Kerr frequency combs using a generalized mean-field Lugiato–Lefever model," Optics Letters **38** (2012), 10.1364/ol.38.000037.

[5] Hossein Taheri, Andrey B. Matsko, and Lute Maleki, "Optical lattice trap for Kerr solitons," The European Physical Journal D **71** (2017), 10.1140/epjd/e2017-80150-6.

[6] Gregory Moille, Qing Li, Travis C. Briles, Su-Peng Yu, Tara Drake, Xiyuan Lu, Ashutosh Rao, Daron Westly, Scott B. Papp, and Kartik Srinivasan, "Broadband resonator-waveguide coupling for efficient extraction of octave-spanning microcombs," Optics Letters **44**, 4737–4740 (2019).
